# Highly Conductive Graphene/Ag Hybrid Fibers for Flexible Fiber-Type Transistors

**DOI:** 10.1038/srep16366

**Published:** 2015-11-09

**Authors:** Sang Su Yoon, Kang Eun Lee, Hwa-Jin Cha, Dong Gi Seong, Moon-Kwang Um, Joon-Hyung Byun, Youngseok Oh, Joon Hak Oh, Wonoh Lee, Jea Uk Lee

**Affiliations:** 1 Composites Research Division, Korea Institute of Materials Science (KIMS), Changwon, 51508, Republic of Korea; 2 Department of Chemical Engineering, Pohang University of Science and Technology (POSTECH), Pohang, 37673, Republic of Korea; 3 Center for Carbon Resources Conversion, Korea Research Institute of Chemical Technology (KRICT), Daejeon 34114, Republic of Korea

## Abstract

Mechanically robust, flexible, and electrically conductive textiles are highly suitable for use in wearable electronic applications. In this study, highly conductive and flexible graphene/Ag hybrid fibers were prepared and used as electrodes for planar and fiber-type transistors. The graphene/Ag hybrid fibers were fabricated by the wet-spinning/drawing of giant graphene oxide and subsequent functionalization with Ag nanoparticles. The graphene/Ag hybrid fibers exhibited record-high electrical conductivity of up to 15,800 S cm^−1^. As the graphene/Ag hybrid fibers can be easily cut and placed onto flexible substrates by simply gluing or stitching, ion gel-gated planar transistors were fabricated by using the hybrid fibers as source, drain, and gate electrodes. Finally, fiber-type transistors were constructed by embedding the graphene/Ag hybrid fiber electrodes onto conventional polyurethane monofilaments, which exhibited excellent flexibility (highly bendable and rollable properties), high electrical performance (*μ*_h_ = 15.6 cm^2^ V^−1^ s^−1^, *I*_on_/*I*_off_ > 10^4^), and outstanding device performance stability (stable after 1,000 cycles of bending tests and being exposed for 30 days to ambient conditions). We believe that our simple methods for the fabrication of graphene/Ag hybrid fiber electrodes for use in fiber-type transistors can potentially be applied to the development all-organic wearable devices.

Wearable electronic materials, such as electronic textiles (e-textiles), have recently attracted great interest because they present exciting possibilities for interfacing computers/processors, sensors, displays, and energy devices with the human body^1^,^2^,^3^. Recently, wearable devices, fabricated by embedding rigid electronic units into clothes, have been introduced and have received a great deal of scientific and social interest^4^,^5^. To truly realize wearable smart electronics, however, every component in the electronic units should be flexible, lightweight, and directly integrated into three-dimensional textile structures.

In recent years, there have been many attempts to integrate electronic functions into fibers, yarns, and fabrics by using various new materials such as conducting polymers^6^,^7^,^8^, carbon-based nanomaterials^9^,^10^,^11^, and metal nanostructures^12^,^13^ as well as their hybrids^14^,^15^,^16^. Among these materials, two-dimensional graphene and its hybrid materials have been considered promising candidates for electronic components of next-generation e-textiles due to their high mechanical flexibility, electrical conductivity, huge specific surface area, and processing possibilities^17^,^18^,^19^,^20^. For example, high-performance graphene fibers have been fabricated; the one-dimensional shape of the fiber can translate the unique properties of graphene nanocomponents into the macroscopic structure needed for flexible and wearable electronic applications. Macroscopic graphene fibers and ribbons were prepared into flexible counter electrodes by using wet-spinning methods to produce wire-shaped, dye-sensitized solar cells^21^,^22^,^23^. Graphene hybrid fibers with unique hierarchical structures were also fabricated by hydrothermal methods and used as flexible, lightweight electrodes for efficient fiber-based electrochemical supercapacitors^24^,^25^,^26^. The aforementioned studies have shown the high potential of graphene and its hybrid fibers as promising candidates for flexible and conductive electrodes in wearable energy devices. However, surprisingly, graphene fibers have not yet been explored for use in flexible transistor applications, which are one of the most important components in wearable devices such as biomedical sensors, wearable displays, and computers.

In this work, we report the fabrication of flexible fiber-type field-effect transistors using graphene/Ag hybrid fibers as highly conductive and flexible electrodes. The flexible and nanoporous graphene fibers were prepared by the wet-spinning of giant graphene oxide and a modified wet-drawing method. The graphene fibers were then hybridized with Ag nanoparticles to form highly conductive graphene/Ag hybrid fibers, which exhibited a record-high electrical conductivity of up to 15,800 S cm^−1^. As the graphene/Ag hybrid fibers can be easily cut and placed onto flexible substrates by simply gluing or stitching, ion gel-gated planar transistors were fabricated by using the hybrid fibers as source, drain, and gate electrodes, and showed electrical characteristics comparable to those of conventional thin-film transistors based on metal electrodes. Finally, fiber-type transistors were constructed by continuously embedding the graphene/Ag hybrid fiber electrodes and transferring the semiconductor/ion-gel dielectric layers onto conventional polyurethane monofilaments, and exhibited excellent flexibility (highly bendable and rollable properties) and high electrical performance (*μ*_h_ = 15.6 cm^2^ V^−1^ s^−1^, *I*_on_/*I*_off_ > 10^4^ in N_2_ atmosphere). Furthermore, the fiber-type transistors showed outstanding stability in terms of device performance (the electrical performance was stable after 1,000 cycles of bending tests and being kept for 30 days outside the glove box).

## Results

### Preparation and characterization of graphene fibers

As a starting material, giant graphene oxide (GGO) sheets were prepared because the large size of the constituent graphene sheets is one of the key factors for the formation of high-performance graphene fibers with both high mechanical strength and electrical conductivity^27^. GGO sheets were synthesized via the microwave-assisted expansion of acid-treated graphite followed by the oxidation and exfoliation of the expanded graphite using previously reported methods (Figure S1)^28^,^29^. GGO sheets have good solubility in water because of their numerous oxygen-containing functional groups, and the GGO solution exhibits excellent liquid crystalline behaviors at concentrations below 10 mg mL^−1^ (Figure S2). Therefore, the GGO layer could be easily aligned under shear force as it passed through a capillary needle during the wet-spinning process^30^,^31^. The average lateral size of the GGO sheets was 56 μm, with a standard deviation of 20 μm, and their thickness measured by the height profile of atomic force microscopy (AFM) analysis was 0.8 nm, indicating that the obtained GGO sheet was a monolayer (Figure S2).

**Figure 1:**
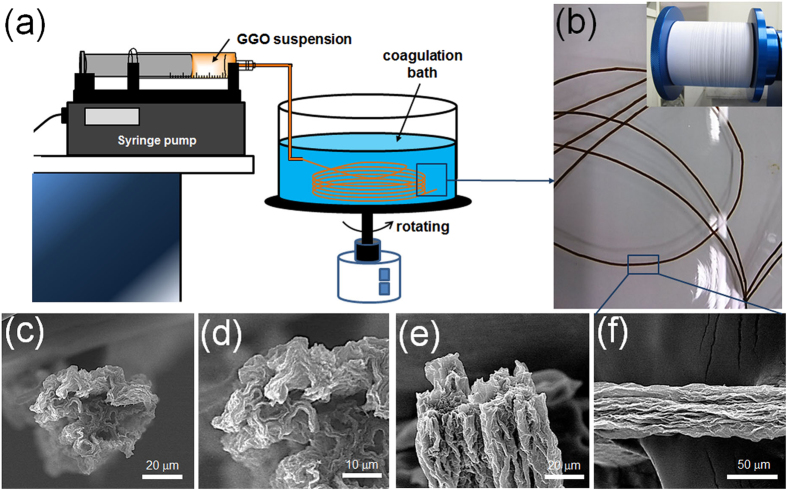
Preparation and microstructures of GGO fibers. (**a**) Schematic of the apparatus used for wet-spinning of GGO fibers. (**b**) Photograph of GGO gel fibers in a CTAB coagulation bath. The inset shows a photograph of the collected as-spun GO fibers. SEM images of the (**c**,**d**) cross-section at different magnitudes, (**d**) fracture surface, and (**e**) outer surface of the GGO fibers spun in a CTAB coagulation bath.

To continuously produce high-performance graphene fibers, the aqueous 10 mg mL^−1^ GGO solution was loaded in a plastic syringe and injected into the rotating coagulation bath with a digital syringe pump (Fig. 1a). Compared with the fibers obtained by various wet-spinning coagulation systems such as non-solvent precipitation^32^, dispersion destabilization using a base or oppositely charged molecules^33^, and coordinative cross-linking using divalent cations^29^, the GGO fibers coagulated in hexadecyltrimethyl ammonium bromide (CTAB) solution (0.5 mg mL^−1^) were the most stable and mechanically robust. Representative scanning electron microscopy (SEM) images and stress-strain curves of the GGO fibers that were spun using the coagulation baths are compared in Figs 1c–e and S3. The GGO fibers spun from the CTAB coagulation bath had round sections and densely ordered lamellar structures due to the electrostatic interactions between the GGO nanosheets and CTAB molecules, which improve the mechanical properties, whereas the other fibers exhibited crumbled morphologies with voids. Therefore, the CTAB aqueous solution was selected as the coagulation solvent for this experiment.

**Figure 2:**
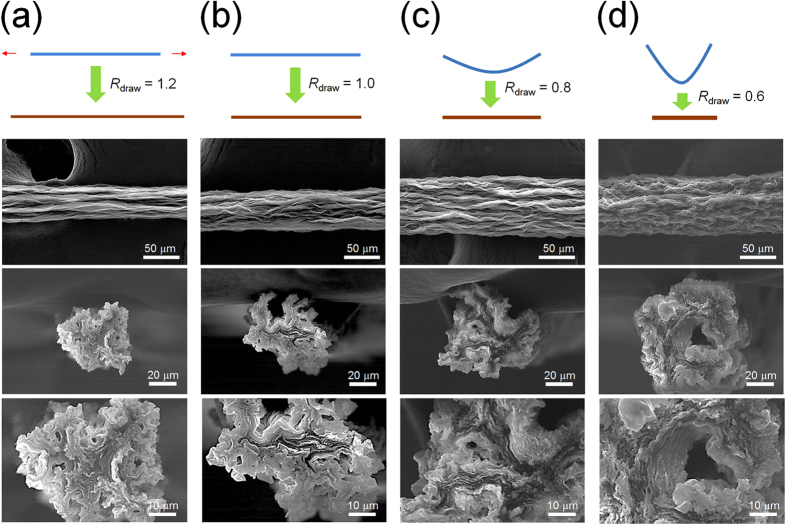
Wet-drawing of GGO fibers. SEM images of the outer surface and cross-section of GGO fibers with a drawing ratio of (**a**) 1.2, (**b**) 1.0, (**c**) 0.8, and (**d**) 0.6, where the drawing ratio is defined as the ratio of the drying end distance to the original length of the gel fibers.

After attaining continuous and stable spinning conditions, such as the appropriate GGO concentration and injection rate, the rotation speed of the coagulation bath, and the width of the syringe outlet (described in the Experimental Section), we focused on the wet-drawing process of the coagulated gel-state fibers to tailor the surface morphology and mechanical properties of the graphene fibers. Because the free-standing gel fibers shrink substantially in a few seconds when removed from the coagulation bath, it is necessary to hold the fibers’ ends during the drying process to maintain the length and morphology. In this process, the wet-drawing system was simply realized by adjusting the distance between the fixtures that clamp each end of the gel fibers; for example, increasing the clamping distance beyond the original length of the gel fiber yielded stretched GGO fibers. Figure 2 displays the change in the cross-section and surface morphologies of the GGO fibers as the drawing ratio was modified, where the drawing ratio (*R*_draw_ = *L*/*L*_0_) is defined as the ratio of the drying end distance (*L*) to the original length of gel fibers (*L*_0_). The diameter and morphologies of the GGO fibers significantly changed as the drawing ratio decreased. The solid fiber resulting from the highest drawing ratio (*R*_draw_ = 1.2) had the smallest diameter of 43 μm and a compact structure, where the GGO sheets were stacked densely with local alignments. The outer surface of the GGO fibers showed wrinkles oriented along the fiber axis -direction and a smooth morphology, which are favorable for improving the mechanical strength. As the drawing ratio decreased, the GGO fibers showed an increased diameter and rougher surface structures. The GGO fiber obtained from the smallest drawing ratio (*R*_draw_ = 0.6) had several pores of a few micrometers in diameter on both the cross-section and the fiber surface as well as extensive random wrinkles, which may be beneficial to the flexibility of the graphene fibers and the embedding of nanoparticles on the fiber surface for the formation of new hybrid materials. When the interlayer microstructures of the GGO fibers were also investigated by analyzing the X-ray diffraction (XRD) pattern of the GGO fibers with the different drawing ratios (Figure S4), the interlayer distance specified by the *d* spacing distance of the (002) plane (*d*_002_) increased from 6.8 Å to 8.1 Å, 9.8 Å, and 12.0 Å as the drawing ratio decreased from 1.2 to 1.0, 0.8, and 0.6, respectively.

To obtain graphene fibers with high mechanical strength and electrical conductivity, GGO fibers have been reduced using various methods such as chemical reduction using hydroiodic acid (HI)^34^ and hydrazine vapor^35^ and by thermal treatment^36^. To maintain the microstructure of the as-prepared GGO fibers, the reduction processes were carried out by attaching the ends of the fibers to the polyethylene terephthalate (PET) frame. Among the applied reduction methods, the chemical reduction by an HI aqueous solution was adopted because the resultant graphene fibers maintained a similar surface morphology to that of the GGO fibers and showed the highest electrical conductivities (Figures S5 and S6). By contrast, the graphene fibers reduced by hydrazine vapor and thermal treatment exhibited slightly altered morphologies and lower conductivities. The diameter of the reduced GGO fibers decreased by approximately 10 μm due to the elimination of the oxygen-containing functional groups of the GGO sheets.

The mechanical and electrical properties of the reduced GGO fibers and the drawing ratios are summarized in Figure S6 and Table 1. The electrical conductivities slightly increased with the drawing ratios because of the denser packing driven by the decreased diameter of the fiber. The mechanical properties of the graphene fibers were more directly influenced by the drawing ratios. Both the ultimate tensile strength (UTS) and Young’s modulus increased up to 240 MPa and 12 GPa, respectively, at *R*_draw_ = 1.2 due to the wrinkles oriented along the fiber axis -direction, in which the graphene sheets were aligned and densely packed by the tensile force during the drying process. By contrast, the graphene fibers obtained when *R*_draw_ = 0.6 had a much lower UTS because of the larger inter-sheet distance. However, the fibers could endure a substantial tensile strain of up to 7% before fracture because of their highly porous and wrinkled structure, which will be useful when further exploring flexible electronic applications.

### Hybridization of Ag nanoparticles on graphene fibers

**Figure 3:**
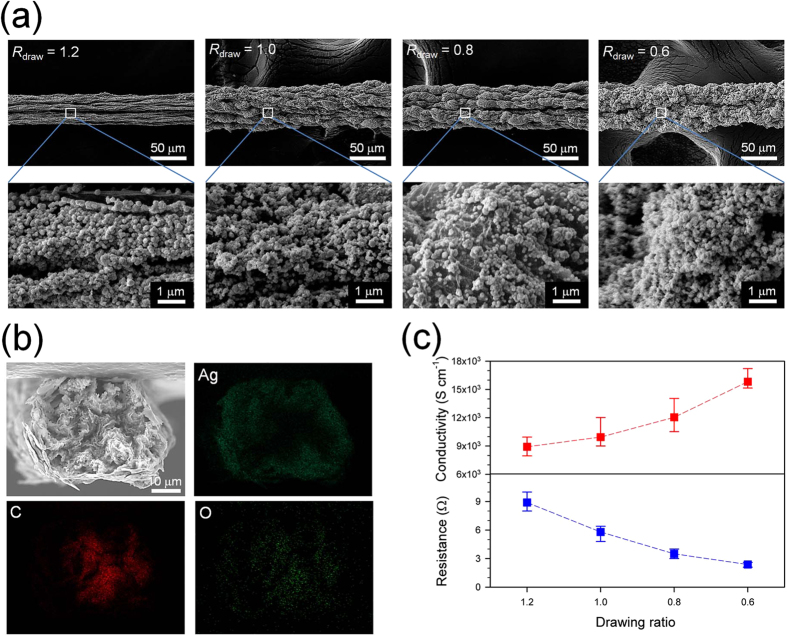
Microstructures and electrical properties of graphene/Ag hybrid fibers. (**a**) SEM images of the outer surface of graphene/Ag hybrid fibers with a drawing ratio of 1.2, 1.0, 0.8, and 0.6. (**b**) Cross-sectional SEM image of the graphene/Ag hybrid fiber (*R*_draw_ = 0.6) and Ag, carbon, and oxygen EDS mapping images. (**c**) Electrical conductivities and resistances of graphene/Ag hybrid fibers as a function of drawing ratios.

To improve the electrical conductivities of the reduced GGO fibers for flexible electronic applications, a silver precursor (AgCF_3_COO in ethanol) was introduced to the graphene fibers and then converted into silver nanoparticles (AgNPs) following previously reported methods that we adapted to our study^37^. The SEM and energy-dispersive spectroscopy (EDS) mapping images of the resultant graphene/Ag hybrid fibers showed that AgNPs with diameters ranging from 100 to 200 nm completely covered the fiber surface, forming a continuous silver shell, and were adsorbed inside the fibers, which is similar to previously reported morphologies of silver-coated fiber mats (Fig. 3a,b). The SEM images and EDS elemental analyses also revealed that more AgNPs were formed on the graphene fibers fabricated using lower drawing ratios (56, 61, 68, and 72 wt% of Ag element for *R*_draw_ = 1.2, 1.0, 0.8, and 0.6, respectively) because the highly porous and wrinkled fibers with a lower drawing ratio had a higher surface area for adsorbing more silver precursors.

By optimizing the reduction conditions of the AgNPs on the conductive graphene fibers, we obtained dramatically enhanced electrical conductivities; with an increase in AgNP content, the mean values of the electrical conductivities of the graphene/Ag fibers increased from 8,920 S cm^−1^ at *R*_draw_ = 1.2 to 15,830 S cm^−1^ at *R*_draw_ = 0.6 (Fig. 3c), which is a record-high electrical conductivity for chemically synthesized graphene-based hybrid fibers and films^38^,^39^. The ultra-high electrical conductivity at *R*_draw_ = 0.6 is attributed to the highly porous and wrinkled structure, which provides sufficient room to incorporate enough AgNPs, together with the intrinsic conductive nature of the reduced graphene fibers.

Because the high flexibility of the conductive fibers is an essential prerequisite for the fabrication of fiber-based wearable electronics, we investigated the effect of bending the graphene/Au hybrid fibers on their electrical properties. Figure S7 displays the change in the electrical resistance of the hybrid fibers as the bending radius (*r*) was decreased. The bendability of the hybrid fibers with lower drawing ratios was superior to that of the hybrid fibers with higher drawing ratios. The electrical resistance of the hybrid fibers with *R*_draw_ = 1.0 and 1.2 showed an abrupt increase at *r* = 1.5 mm, whereas the fiber with *R*_draw_ = 0.6 exhibited no difference in resistance, even for low values (*r* = 1 mm). In addition, when we monitored the change in the resistance of the hybrid fiber obtained when *R*_draw_ = 0.6 during cyclic tests (repeatedly bending to *r* = 2 mm), the electrical resistance remained stable and only varied negligibly in both the bent and the straight state over 1,000 cycles (Figure S7e).

### Planar transistor devices using hybrid fiber electrodes

**Figure 4:**
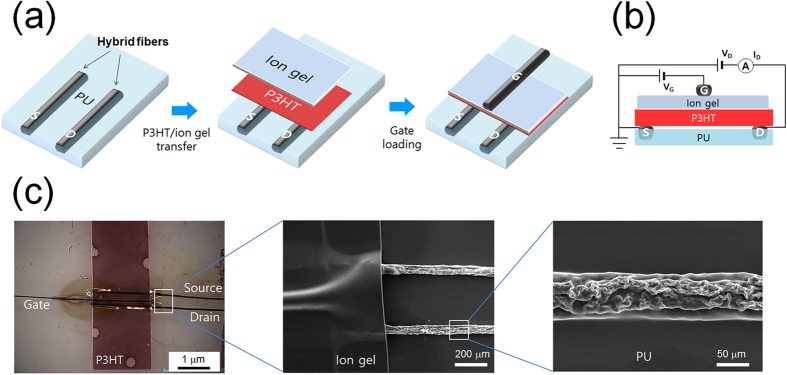
Preparation of a planar OTFT device using hybrid fiber electrodes. (**a**) Schematic of the fabrication process of a planar OTFT device based on the hybrid fiber electrodes. (**b**) Cross sectional schematic of the planar OTFT device. (**c**) Optical microscopy image of the planar OTFT device and magnified SEM images of the fiber electrodes.

Encouraged by the outstanding electrical conductivity and electromechanical stability of the graphene/Ag hybrid fibers, we fabricated organic thin-film transistors (OTFTs) by using the hybrid fibers as electrodes because the most important and widely used component in both conventional electronics and wearable devices is the transistor, which forms the basis for complex circuitry. Although a number of flexible conductors have been successfully developed by using carbon nanotubes^40^,^41^, the realization of flexible devices based on carbon nanomaterials and their hybrids is much more difficult because all the components of the devices should be flexible and maintain high-quality interfaces together. The OTFT devices were fabricated in a bottom-contact, top-gate architecture with the hybrid fiber electrodes, as schematically depicted in Fig. 4a. Figure S8 shows photographs of the fabrication procedure, and a more thorough description is given in the Experimental Section. The first step was to embed the graphene/Ag hybrid fibers as source and drain (S/D) electrodes in an as-casted polyurethane (PU) layer, in a way that would expose the top part of the fiber electrodes out of the PU layer and allow contact with the upper layer upon drying of the PU substrate. We also chose poly(3-hexylthiophene) (P3HT) as the solution-processable p-channel semiconductor because it is one of the most widely used polymer semiconductors. The direct spin coating of P3HT on the PU substrate was not suitable because the PU layer was damaged and the aligned fiber electrodes could be distorted by the chloroform solvent during the spin coating process. To avoid this problem, we used an elastic ion gel layer based on poly(vinylidene fluoride-*co*-hexafluoropropylene), P(VDF-HFP), and the ionic liquid 1-ethyl-3-methylimidazolium bis(trifluoromethylsulfonyl)amide ([EMI][TFSA])^42^ as both the high capacitance gate dielectric layer and the mechanically robust transporter for the channel material (P3HT). After the successive spin coating of the ion gel and the P3HT layers on a glass slide, the double layer was cut with a razor blade and inversely transferred onto the PU substrate for direct contact between the P3HT layer and the fiber electrodes. This procedure provided a convenient and solvent-free route to simultaneously incorporate the active channel and the ion gel layers in the transistors without any contamination of each component. The fabrication of planar-structured transistors was completed by simply laying another hybrid fiber on the ion gel layer as a top gate electrode and then applying a new ion gel layer on top to enclose the device and induce contact between the fiber gate electrode and the dielectric layer. A reference device was also prepared by using the reduced graphene oxide fibers as the S/D and -gate electrodes instead of the graphene/Ag hybrid fibers to elucidate the effect of the electrical conductivities of the fiber electrodes on the device performance of the transistors.

Figure 4c displays the optical microscopy (OM) and SEM images of the transistor device developed in this study, where the hybrid fiber-based source-drain electrodes were well aligned with each other. The thickness of the PU substrate, of P3HT, and of the ion gel layer was 50 μm, 60 nm, and 11 μm, respectively. The clear hybrid morphology at the center and the indistinct edges of the S/D fiber electrodes in the magnified SEM image confirm that the major part of the graphene/Ag hybrid fiber was embedded in the PU substrate and that only the top part was exposed for contact with the P3HT layer. The channel length (*L*) was defined as the distance between two aligned S/D fiber electrodes; the distance in this study was 200 ~ 300 μm. The channel width (*W*) is also defined as the width of the transferred P3HT layer, which was 2 ~ 4 mm. The gate electrode fiber was aligned along the S/D electrode direction and sandwiched between ion gel layers.

**Figure 5:**
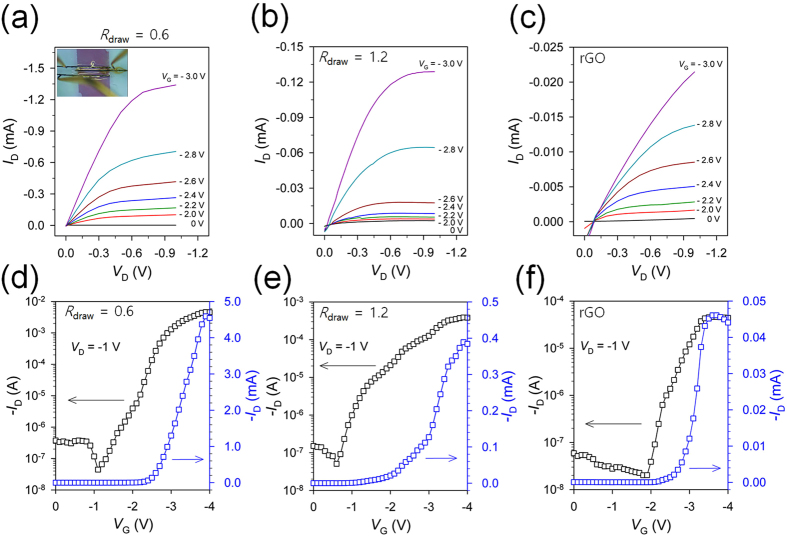
Electrical characteristics of planar transistor devices. (**a**–**c**) *I*_D_–*V*_D_ and (**d**–**f**) *I*_D_–*V*_G_ characteristics of the planar transistors based on graphene/Ag hybrid fiber electrodes with *R*_draw_ = 0.6 or, 1.2 and reduced graphene oxide fiber electrodes, respectively. The inset shows the optical image of a planar transistor device with a channel length of 300 μm and a channel width of 2 mm.

Figure 5a–c displays the typical output characteristics (*I*_D_-*V*_D_, where *I*_D_ is the drain current and *V*_D_ is the drain voltage) of a hybrid fiber (*R*_draw_ = 0.6) electrode-based OTFT at different gate voltages (*V*_G_s). The well-defined gate modulation in the output curves reveals the ohmic contact between the hybrid fiber electrodes and the transferred semiconductor/dielectric layers. The drain current shows a linear behavior at low drain voltages and a saturation behavior in the high voltage regime. The saturation current is greater than 1.3 mA at *V*_G_ = −3 V and *V*_D_ = −1 V as a result of the high conductivity of the hybrid fiber electrodes and the large specific capacitance of the ion gel gate dielectric, which is as large as 9 μF cm^−2^ at low frequency (corresponding to a thickness of 11 μm), as shown in Figure S9^42^. The transistors based on the hybrid fiber electrode with *R*_draw_ = 1.2 and on the reduced graphene fiber electrode also show similar output characteristics, with a lower saturation current of 0.12 mA and 0.02 mA at *V*_G_ = −3 V and *V*_D_ = −1 V, respectively.

To compare the important electrical characteristics of the transistors, such as the charge carrier mobility (*μ*_h_), on/off current ratio (*I*_on_/*I*_off_), and threshold-voltage (*V*_T_), the drain current was measured while sweeping *V*_G_ from 0 V to −4 V at a rate of 33 mV s^−1^ and a constant *V*_D_ value of −1 V. The typical transfer curves with a logarithmic scale (left axis) and a linear scale (right axis) with different fiber electrodes are provided in Fig. 5d–f, and the device parameters are summarized in Table 2. From the slope of the *V*_G_ vs. |*I*_D_| curves obtained for more than five devices, the average field-effect mobility was estimated in the linear regime (*V*_D_ = −1 V) from the following equation^43^:


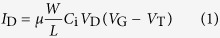


where *I*_D_ is the drain current, *μ* is the field-effect mobility, *C*_i_ is the specific capacitance of the dielectric, *V*_D_ is the drain voltage, *V*_G_ is the gate voltage, *V*_T_ is the threshold voltage, and *W* and *L* are the channel width and length, respectively. All three devices showed low *V*_T_ values around −2.5 ~ 2.7 V. Despite the manual fabrication process that involved embedding the S/D electrodes, transferring the semiconductor/dielectric layers, and applying the top gate, the transistors made of the hybrid fiber electrodes showed a reasonably high *I*_on_/*I*_off_ of 10^4^ ~ 10^5^. However, the transistor made of rGO fiber electrodes exhibited a very low on-current (~10^−5^ A), which is two orders of magnitude lower than those of the device made of hybrid fiber electrodes, leading to a low on/off current ratio (~10^3^). The average hole mobility was calculated to be 20.4 and 4.4 cm^2^ V^−1^ s^−1^ for hybrid fiber-based transistors prepared at *R*_draw_ = 0.6 and 1.2, respectively, which are much higher than those reported in other P3HT-based transistors gated with conventional dielectrics (0.1 ~ 0.01 cm^2^ V^−1^ s^−1^)^44^,^45^ but are comparable to other recent results on electrolyte-gated polymer transistors^46^. It has been speculated that the high mobility value in this result is due to the penetration of ions from the ion gel dielectric into the active channel that fills the carrier traps and acts as a dopant in the P3HT film^47^,^48^. By contrast, the transistor made of rGO fiber electrodes shows a much lower mobility of 0.4 cm^2^ V^−1^ s^−1^ because of the lower on-current and slightly higher threshold voltage. These results indicate that the use of highly conductive hybrid fiber electrodes provides a reliable alternative to the deposition of metal electrodes for the preparation of high-performance transistors.

### Fiber-type transistor devices using hybrid fiber electrodes

**Figure 6:**
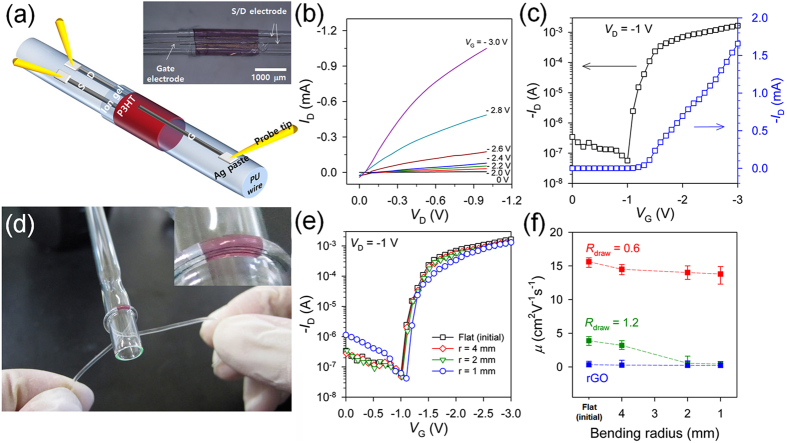
Electrical characteristics and flexibility of fiber-type transistor devices. (**a**) Schematic illustration and optical image of a fiber-type transistor based on graphene/Ag hybrid fiber electrodes. (**b**) *I*_D_–*V*_D_ and (**c**) *I*_D_–*V*_G_ characteristics of the fiber-type transistors based on hybrid fiber electrodes with *R*_draw_ = 0.6. (**d**) Photograph of a fiber-shaped transistor wrapped around a glass rod (*r* = 2 mm). The inset shows the bent hybrid fiber electrodes along the curvature of the glass rod. (**e**) Changes in the transfer curves of the fiber transistor devices based on hybrid fibers (*R*_draw_ = 0.6) at different bending radii. (**f**) Changes in the hole mobilities of the fiber transistor devices based on hybrid fibers (*R*_draw_ = 0.6 and 1.2) and on the rGO fiber electrodes at different bending radii.

We further applied the graphene/Ag hybrid fibers as electrodes for fiber-type flexible transistors. The fiber transistor was fabricated by first forming the S/D electrodes onto a flexible PU monofilament (diameter ~1 mm), the top of which was slightly dissolved with THF to embed the graphene/Ag hybrid fibers instead of using the as-casted PU substrate (Figure S10). After transferring the P3HT/ion gel double layer, cutting away the uncovered region of double layer induces a complete contact between the S/D electrodes on the PU monofilament and the P3HT/ion gel double layer. The application of the gate electrode fiber over the channel, followed by covering the filament with new ion gel layer for protection from exfoliation results in the formation of an electrolyte-gated transistor device on the conventional PU filament. Each electrode was connected to Cu wires with silver paste to measure the electrical characteristics of the fiber-type flexible transistors during the bending. The optical microscopy image indicates that the S/D and G electrode fibers were well aligned along the PU filament direction and that the P3HT channel and ion gel layers successively covered the PU filament (Fig. 6a). The channel length, defined as the distance between two aligned S/D fiber electrodes on the PU filament, was approximately 200 ~ 300 μm, and the channel width, defined as the width of the transferred P3HT layer, was 2 ~ 4 mm.

Figure 6 displays the typical *I*_D_-*V*_D_ and *I*_D_-*V*_G_ characteristics of a fiber transistor made using hybrid fibers (*R*_draw_ = 0.6), which shows a gate modulation of the drain current that is similar to that of the planar OTFT devices. The average transistor characteristics (measured for more than 5 devices) include a hole mobility value of 15.6 cm^2^ V^−1^ s^−1^, an on/off current ratio of 10^4^, and a threshold voltage of 1.2 V. These values are comparable to those exhibited by the typical curves of planar transistor devices fabricated using the same hybrid fiber electrodes. The decrease in mobility is attributed to an increase in the effective channel length due to the curvature of the PU filament substrate compared to the planar PU film substrate.

Furthermore, the fiber transistor was bendable and could even be rolled up because all the components of the transistor, including the PU filament, hybrid fiber electrodes, and P3HT/ion gel thin layers, were very flexible and mechanically stable (Fig. 6d). Figure 6e shows the transfer curves of the fiber-type transistors with hybrid fiber electrodes (*R*_draw_ = 0.6) obtained at different bending radii. The transfer curves of the fiber transistor maintained a stable transistor behavior at all bending radii, and the device performance (*μ*_h_, *I*_on_/*I*_off_, and *V*_T_) calculated from the transfer curves showed negligible change during the bending test (Fig. 6f). By contrast, the transistors fabricated using hybrid fiber electrodes with a higher drawing ratio (*R*_draw_ = 1.2) showed highly unstable device performance; a considerable decrease in hole mobility was observed when the bending radius was decreased to 2 mm, which could be caused by the abrupt increase in the electrical resistance of the fiber electrodes during bending, as shown in Figure S7. The transfer curves of the fiber-type transistors prepared using hybrid fibers with *R*_draw_ = 1.2 and rGO fiber electrodes, which were obtained at different bending radii, are shown in the supporting information (Figure S11).

In addition, we measured the changes in the transfer curve and hole mobility of the fiber-type transistor after the cyclic bending test, stretching test, and storage in ambient conditions for a long time because the stability of the device performance is critical for practical applications (Figures S12 and S13). Remarkably, after bending the device to a radius of 2 mm for 1,000 cycles, the fiber transistor with hybrid fiber electrodes (*R*_draw_ = 0.6) showed transfer curves that were similar to the original curve and a small decrease in the hole mobility (11.7 cm^2^ V^−1^s^−1^). More importantly, the mobility value of the devices remained nearly unchanged when the device was stretched to 5% and left to stand in air for 30 days. The outstanding durability of the device originates from the high mechanical flexibility of the hybrid fiber electrodes and the closely packed device structure of the fiber transistor due to the ion gel capping layers.

## Discussion

In summary, we prepared highly conductive and flexible graphene/Ag hybrid fibers and used them as electrodes for planar and fiber-type transistors. By changing the conditions of the wet-spinning and wet-drawing processes of the graphene fibers, we could tune their mechanical properties, flexibilities, and electrical conductivities. The graphene hybrid fiber obtained with the lowest drawing ratio (*R*_draw_ = 0.6) exhibited excellent flexibility and a record-high electrical conductivity of up to 15,800 S cm^−1^. Due to the high conductivity and flexibility of the graphene hybrid fibers, the planar and fiber-type transistor devices fabricated using those graphene hybrid fibers as source, drain, gate electrodes showed electrical characteristics that are superior to those of conventional thin-film transistors based on metal electrodes as well as excellent flexibility (bendable and rollable). Furthermore, the fiber-type transistor showed an outstanding stability in terms of device performance (the electrical performance was stable after 1,000 cycles of bending at *r* = 2 mm and being kept for 30 days outside the glove box), which originates from the high mechanical flexibility of the hybrid fiber electrodes and the closely packed device structure of the fiber transistor due to the ion gel capping layers.

## Methods

### Materials

All materials were purchased from Sigma-Aldrich, except for the natural graphite flakes (~500 μm flake, Smajung cng. Inc.), soluble polyurethane (Estane 5700 TPU, Lubrizol Inc.), polyurethane wire (diameter ~1 mm, Hae Kwang Inc.) and Poly(3-hexylthiophene) (MW = 50 K, Rieke Metals Inc.).

Giant graphene oxide sheets were synthesized via microwave-assisted expansion of expandable graphite followed by oxidation and exfoliation of expanded graphite using the previously reported methods^28^,^29^. Briefly, natural graphite flakes (5 g), 98% sulfuric acid (200 mL) and fuming nitric acid (60 mL) were stepwisely added into a 500 mL flask and the mixture was stirred for 24 h at room temperature. The mixture was then poured slowly into 1 L of water and filtered and washed using water for several times to collect solid graphite. After drying at 60 °C for 24 h, acid-treated graphite was sealed in a glass vial and purged with high purity nitrogen for 2 h. The vial was then heated in a microwave oven (Daewoo Electronics, TMW-1100EK, 60 Hz) for 3 s. In the second step, 5 g of expanded graphite powder, 300 mL sulfuric acid, 4.2 g K_2_S_2_O_8_ and 6.2 g P_2_O_5_ were added successively into a 500 mL flask and the mixture was kept at 80 °C for 5 h. After cooling to room temperature, the mixture was diluted with 2L water and vacuum-filtered and washed with water. The solid was dried in air at room temperature for 2 days. In the last step, the preoxidized expanded graphite was added into 200 mL concentrated H_2_SO_4_ (0 °C), and then 15 g KMnO_4_ was added slowly under continuous stirring. After then, the mixture was heated to 35 °C and stirred for 2 h. The mixture was then diluted with 2L water, followed by dropwise addition of 20 mL 30% H_2_O_2_. The mixture was left undisturbed for 2 days and the nearly clear supernatant was decanted. Using centrifugation washing method, the precipitate was repeatedly washed with water, 1M HCl solution and water successively. Trace amounts of ions are then removed by dialysis against deionized water for 3 days, using tubing with a 12,000 MW cut off (Spectrum Laboratories, Inc.). The final graphene oxide solution is then concentrated, yielding a very viscous, brownish transparent solution with concentration of 0.4% (w/w).

### Graphene/Ag hybrid fiber preparation

The 10 mg mL^−1^ of GGO spinning dopes were loaded in a plastic syringe (24 G, width of 292 μm) and injected into the rotating CTAB coagulation bath of 0.5 mg mL^−1^ with the injection pump (injection rate of 100 μL/min). After 30 min immersion in coagulation bath, the GGO fibers were transferred into water bath to wash away the residual coagulation solution, and then dried under controlled tension at room temperature. The wet-drawing of gel fibers in the drying process was realized by adjusting the fixtures distance which clamp the each end of the gel fibers. The dried GGO fibers were chemically reduced by dipping the fibers (under tension) in HI aqueous solution (47%) at 90 °C for 12 h. For comparison, the reduction was carried out by exposing the fibers to hydrazine vapor (100%) at 90 °C for 12 h and thermal annealing at 800 °C for 4 h. After cooling to room temperature, the fibers were washed with water and ethanol in succession and dried at 100 °C under vacuum for 12 h.

To fabricate graphene/Ag hybrid fibers, the reduced GGO fibers were dipped (under tension) into a AgCF_3_COO solution in ethanol (15 wt%) for 30 min. After 5 min of evaporation in air, the fibers were immersed into the hydrazine hydrate (50%) in a mixture of ethanol/water (1:1 in volume ratio) for 5 min to reduce the adsorbed Ag ions to AgNPs. The fibers were washed with water and dried at room temperature under vacuum for 12 h. The above procedures were repeated to obtain the desired amount of AgNPs on the graphene fibers.

### Capacitance measurement

Capacitance measurements were carried out using a Biologic VSP-300 potentiostat at room temperature. Metal-ion gel-metal (MIM) capacitors were created and the experiments were conducted over the frequency range of 1–10^4^ Hz with an AC amplitude of 10 mV.

### Fabrication of Planar Transistor Devices

Polyurethane (PU) substrates were created by dissolving PU in dimethylformamide (DMF) at a concentration of 100 mg mL^−1^ and casting onto a slide glass. Before drying the PU substrate, as-prepared graphene/Ag hybrid fiber was embedded onto the PU layer to expose the top part of the fiber. The second hybrid fiber was successively embedded onto the PU layer and located 200 ~ 300 μm away from the first embedded hybrid fiber, leading to the well aligned hybrid fiber-based source-drain electrodes.

Silicon (Si) wafer was cleaned by piranha solution to remove any organic contamination and treated with oxygen plasma to introduce hydrophilic surface. The ion gel layer was prepared by first codissolving P(VDF-HFP) and the ionic liquid, [EMI][TFSA], in acetone (the weight ratio between the polymer, ionic liquid, solvent was kept to 1:4:7), and then spin coating on the washed Si wafer at 1000 rpm for 1 min. Spin-coated ion gel layers were placed in a vacuum at 70 °C for 24 h to remove the residual solvent. Regioregular P3HT was spin coated at 2000 rpm for 60 s from chloroform solution (10 mg mL^−1^) on ion gel layer. The double layer of ion gel and P3HT was cut with a razor blade, and then inversely transferred onto the PU substrate for direct contact between P3HT layer and the hybrid fiber electrodes. To complete the fabrication of planar-structured transistor devices, the third graphene/Ag hybrid fiber was simply laid on the ion gel layer as a top gate electrode, and then new ion gel layer was applied over the gate for close contact between fiber gate and ion gel layer.

### Fabrication of Fiber-Type Transistor Devices

Flexible polyurethane monofilament (diameter ~1 mm) was used as the substrate for the fabrication of fiber-type transistor devices. First, the PU filament was washed with ethanol and settled on the glass slide by using 3M tape. The top of the PU wire was slightly dissolved with THF solvent and two graphene/Ag hybrid fibers were embedded parallel to each other, leading to the well aligned hybrid fiber-based source-drain electrodes on the PU filament. The double layer of ion gel and P3HT was inversely transferred from Si wafer onto the PU filament and then the uncovered region of the double layer was cut away for direct contact between P3HT layer and the S/D electrodes. To complete the fabrication of fiber-type transistor devices, the third graphene/Ag hybrid fiber was simply laid on the ion gel layer as a top gate electrode, and then new ion gel layer was applied over the gate for close contact between fiber gate and ion gel layer. Each electrode was connected to Cu wires with silver paste to measure the electrical characteristics of the fiber-type flexible transistors during the bending test.

### Characterization

OM and POM observations were performed with a Nikon ECLIPSE LV150N. SEM images were taken on a JEOL JSM5800. AFM images and height profiles of GGO sheets were taken in the tapping mode of Park systems NX10, with samples prepared by spin-coating from aqueous solutions onto freshly cleaned Si/SiO_2_ wafer. XRD patterns were recorded with a Rigaku D/Max-2200 diffractometer using CuKα (λ = 0.154 nm) radiation. The mechanical properties of the graphene fibers were characterized by the uniaxial tensile tests with a dynamic mechanical analyzer (DMA Q800, TA Instruments). Tensile tests were conducted in controlled-displacement mode with preload of 0.01 N, and force was loaded with a displacement ramp rate of 0.05 N min^−1^. The electrical properties of graphene and graphene/Ag hybrid fibers were characterized using two-probe method on an electrical transport properties measurement system composing a Keithley 2100 multiple–function source-meter. The cross-section area of the fiber was measured from the fracture surface of SEM image. The bending test was carried out with a home-made two-point bending device and a high-precision mechanical system. The measurement of the current-voltage characteristics of planar-structured and fiber-type transistor devices were carried out at room temperature in a N_2_-atmosphere glove box using MST-5500B probe station and Keithley 4200-SCS.

## Additional Information

**How to cite this article**: Yoon, S. S. *et al*. Highly Conductive Graphene/Ag Hybrid Fibers for Flexible Fiber-Type Transistors. *Sci. Rep*. **5**, 16366; doi: 10.1038/srep16366 (2015).

## Supplementary Material

Supplementary Information

## Figures and Tables

**Table 1:** Mechanical and electrical properties of reduced GGO fibers at different drawing ratios.

Drawing ratio	Tensile strength [MPa]	Elongation at break [%]	Young’s modulus [GPa]	Conductivity^a^ [S cm^−1^]
1.2	239.3	2.7	12.1	17.0
1.0	202.4	3.2	8.2	16.7
0.8	145.7	4.9	4.8	6.8
0.6	119.4	7.2	2.4	6.0

^a^Electrical conductivities of graphene fibers reduced by HI aqueous solution.

**Table 2:** Average hole-mobilities, on/off current ratios, and threshold voltages of planar OTFT devices and fiber-type transistor devices based on the graphene fiber electrodes.

Transistor type	Fiber electrode	*μ*_h_ [cm^2^ V^−1^ s^−1^]	*I*_on_/*I*_off_	*V*_T_ [V]
Planar device	hybrid fiber (0.6)^a^	20.4	1.0 × 10^5^	−2.5
	hybrid fiber (1.2)	4.4	1.0 × 10^4^	−2.6
	rGO fiber (0.6)	0.4	2.3 × 10^3^	−2.7
Fiber-type device	hybrid fiber (0.6)	15.6	3.4 × 10^4^	−1.2
	hybrid fiber (1.2)	3.4	8.0 × 10^3^	−2.3
	rGO fiber (0.6)	0.3	6.0 × 10^3^	−2.5

^a^The number in parentheses indicates the drawing ratio of the fiber electrode during fiber formation.
