# Supplementary material for: Highly Conductive Graphene/Ag Hybrid Fibers for Flexible Fiber-Type Transistors
Source: Sci Rep. 2015 Nov 9;5:16366. doi: 10.1038/srep16366 (PMC4637867; doi:10.1038/srep16366)
Supplement: Supplementary Information [file srep16366-s1.pdf]

Supplementary information

Highly Conductive Graphene/Ag Hybrid Fibers for Flexible Fiber-Type Transistors

Sang Su Yoon,[1] Kang Eun Lee,[1] Hwa-Jin Cha,[1] Dong Gi Seong,[1] Moon-Kwang Um,[1] Joon Hyung Byun,[1] Youngseok Oh,[1] Joon Hak Oh,[2] Wonoh Lee,[1,]* and Jea Uk Lee[1,3]*

[1] Composites Research Division, Korea Institute of Materials Science (KIMS), Changwon, 51508, Republic of Korea

[2] Department of Chemical Engineering, Pohang University of Science and Technology (POSTECH), Pohang, 37673, Republic of Korea

[3] Center for Carbon Resources Conversion, Korea Research Institute of Chemical Technology (KRICT), Daejeon 34114, Republic of Korea

*Correspondence and requests for materials should be addressed to J.U.L. (email: leeju@krict.re.kr) or W.L. (email:wonohlee@kims.re.kr)

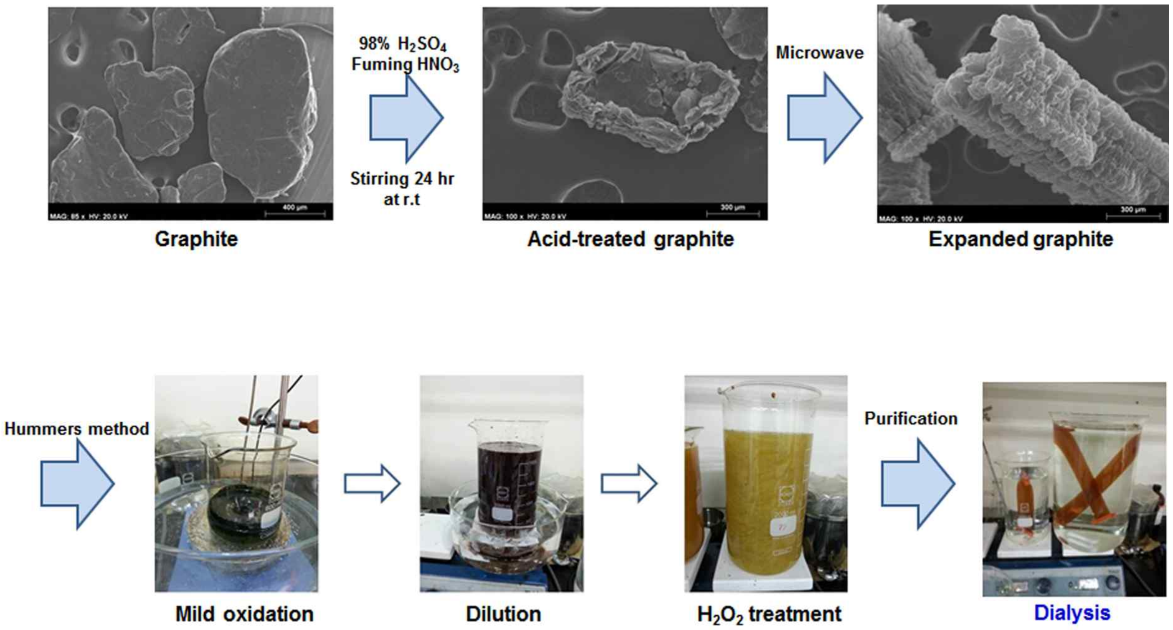

Figure S1 | Synthesis procedure of a giant graphene oxide.

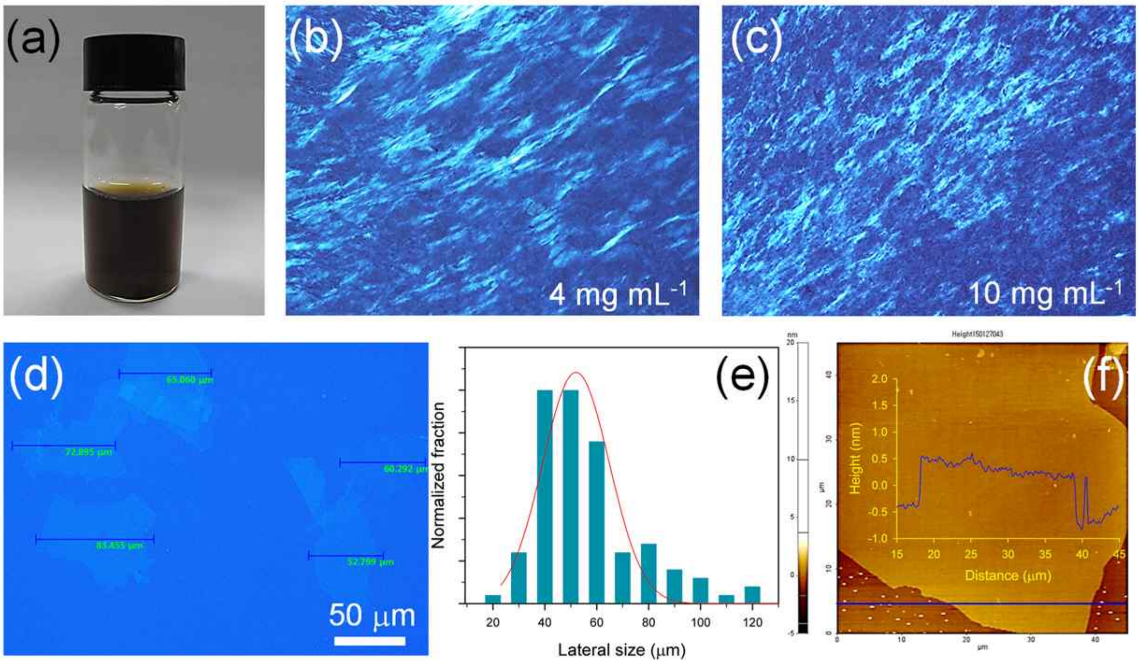

Figure S2 | (a) Photograph of GGO aqueous solution at a concentration of 10 mg mL^{-1}. Representative polarized optical microscopy images of liquid crystalline GGO solutions loaded in the planar cells, at concentrations of (b) 4 mg mL^{-1} and (c) 10 mg mL^{-1}. (d) Optical microscopy image of GGO sheets deposited on silicon wafer. (e) The lateral size distribution of GGO sheets. The irregular sheets are regarded as squares of equal area and the lateral size of sheets as the side length of squares. (f) AFM image of GGO sheet deposited on silicon wafer. The inset shows the height profile derived from the blue line in the AFM image showing the thickness of monolayer GGO sheet is 0.8 nm.

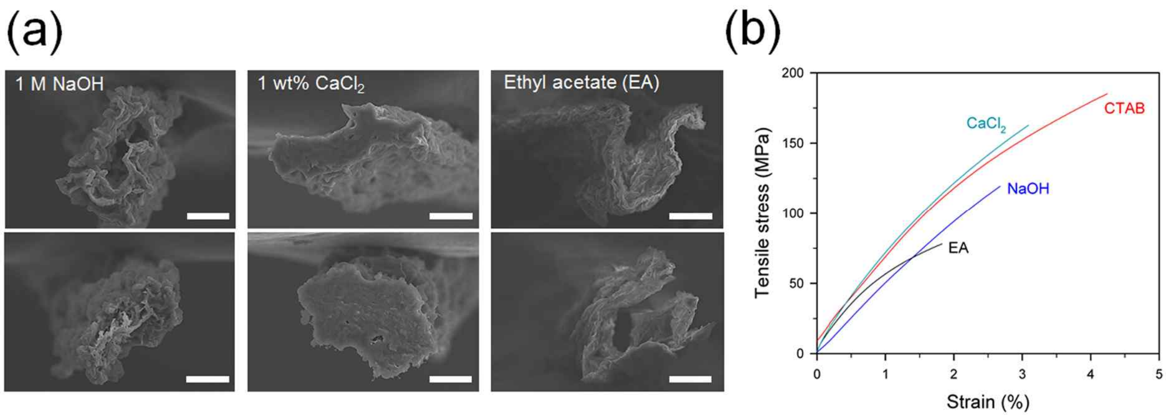

Figure S3 | (a) Cross-sectional SEM images of the GGO fibers spun in NaOH, $CaCl_2$ water solutions, and ethyl acetate solvent (scale bar: 30 μm). (b) Typical strain-stress curves of the GGO fibers spun in each coagulation bath.

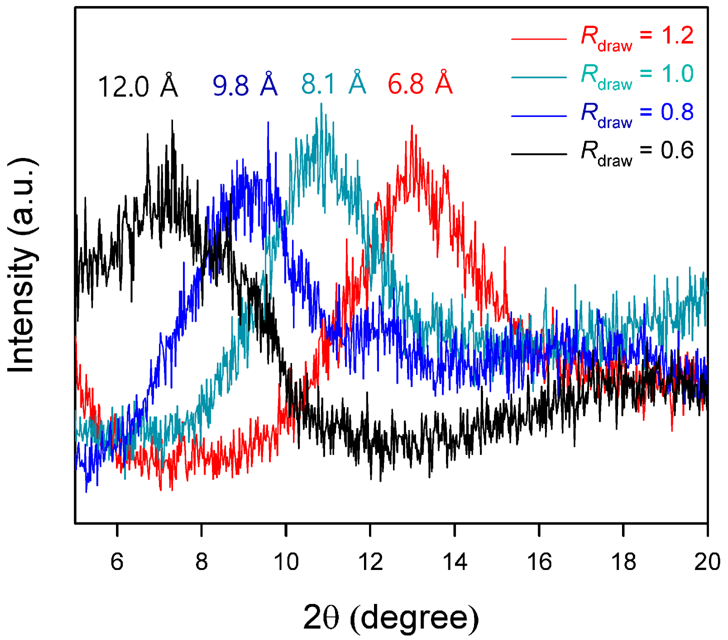

Figure S4 | XRD patterns collected from the GGO fibers with a drawing ratio of 1.2, 1.0, 0.8, and 0.6.

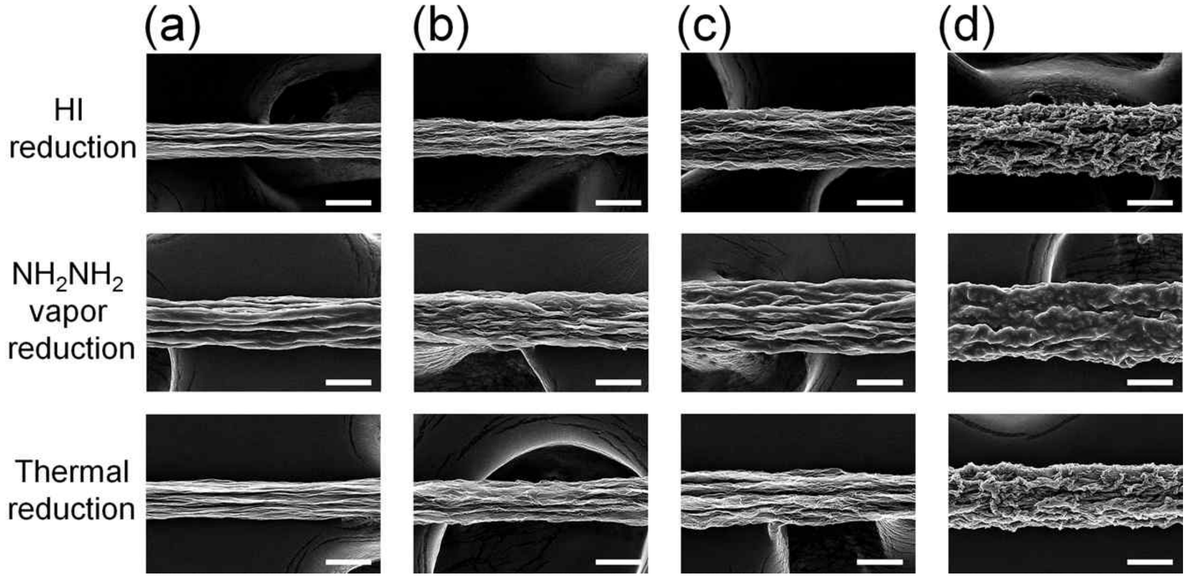

Figure S5 | SEM images of the outer surface of graphene fibers with a drawing ratio of (a) 1.2, (b) 1.0, (c) 0.8, and (d) 0.6, reduced by HI aqueous solution (top), hydrazine vapor (middle), and thermal treatment (bottom). All scale bars are 50 μm.

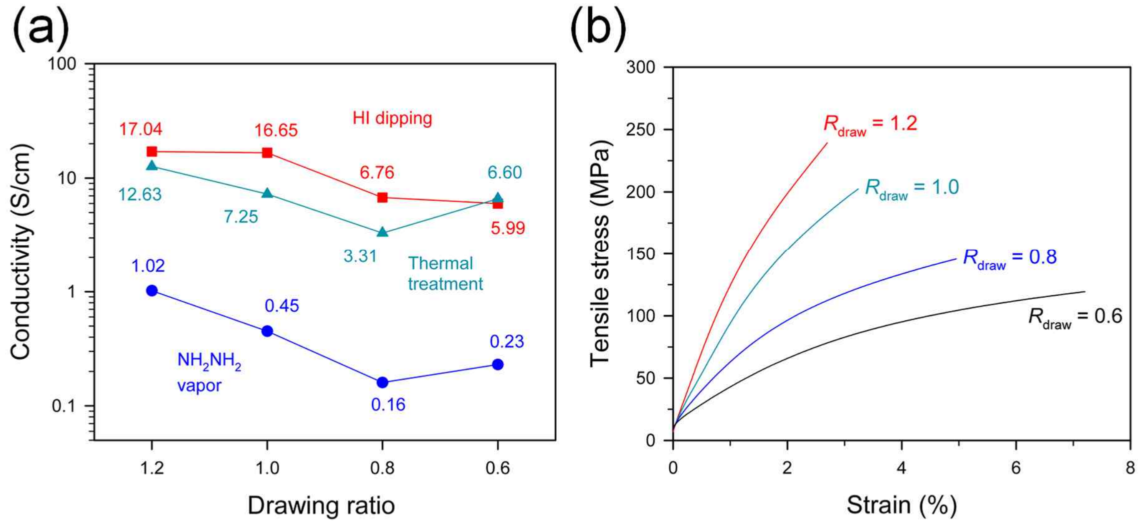

Figure S6 | (a) Electrical conductivities of graphene fibers reduced by HI aqueous solution (red squre), hydrazine vapor (blue circle), and thermal treatment (green triangle) as a function of drawing ratios. (b) Typical strain-stress curves of graphene fibers with a drawing ratio of 1.2, 1.0, 0.8, and 0.6, reduced by HI aqueous solution.

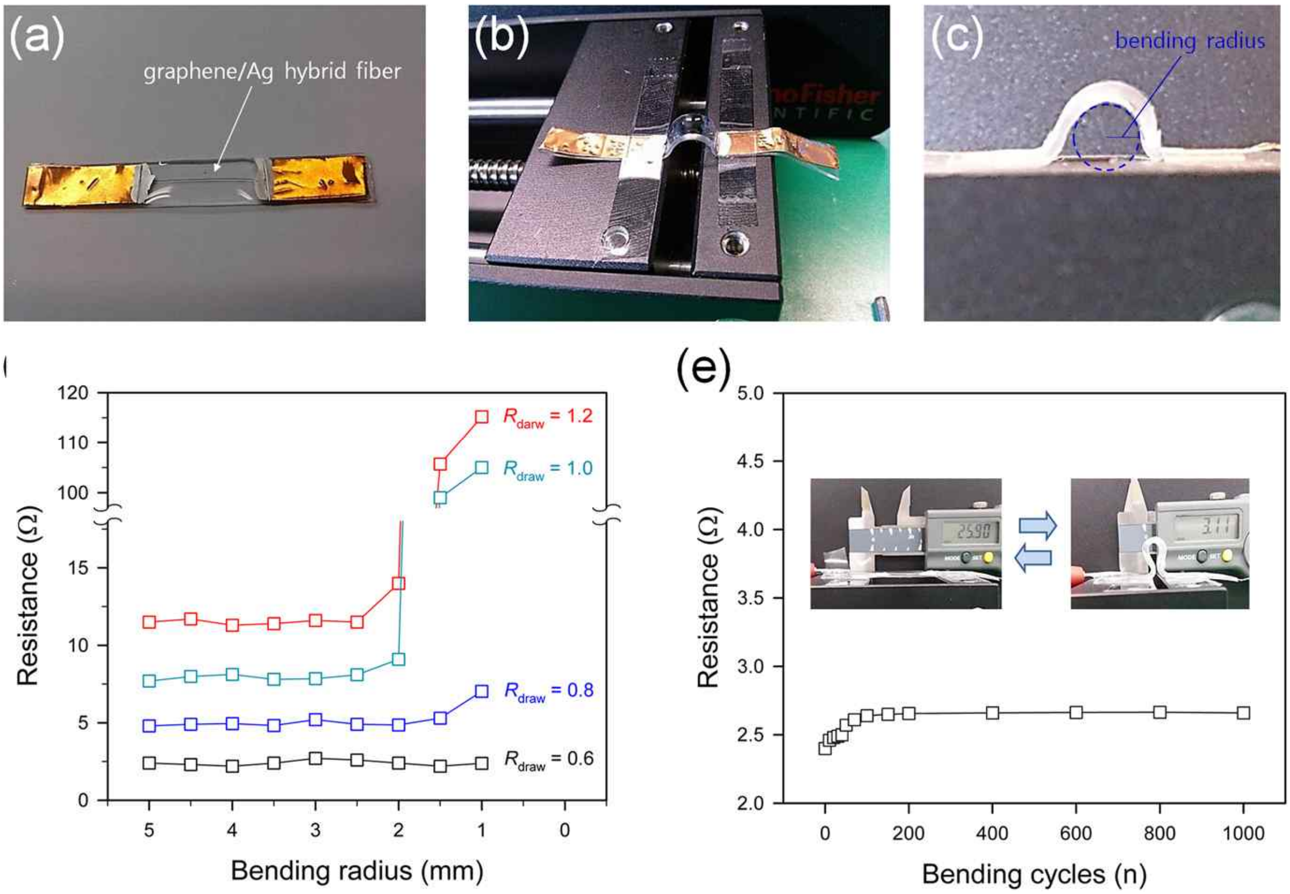

Figure S7 | Photographes of flexible graphene/Ag hybrid fiber (a) embedded in PDMS film, (b) loaded onto automatic bending machine. (c) Side-view image of bended hybrid fiber and bending radius. (d) Electrical resistance change of the graphene/Ag hybrid fibers with a drawing ratio of 1.2, 1.0, 0.8, and 0.6, as a function of bending radius. (e) Electrical resistance change of the graphene/Ag hybrid fiber with R_{draw} = 0.6 depending on the bending cycle. The inset photographs show the straight and bent state of the graphene/Ag hybrid fiber.

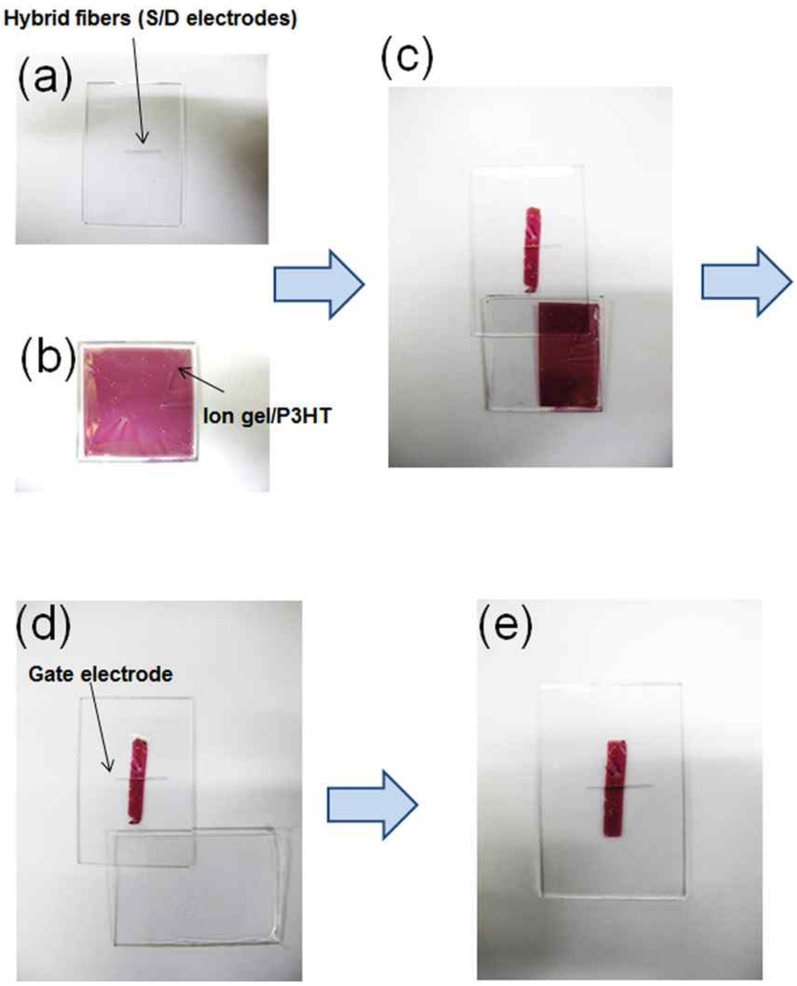

Figure S8 | Digital images of the planar transistor device at different stages during fabrication. (a) Hybrid fiber electrodes (S/D) embedded in a PU substrate. (b) Ion gel/P3HT double layer spin coated onto the glass slide. (c) Double layer inversely transferred onto the S/D electrodes. (d) Gate electrode fiber applied onto the ion gel layer. (e) New ion gel layer covered the gate electrode.

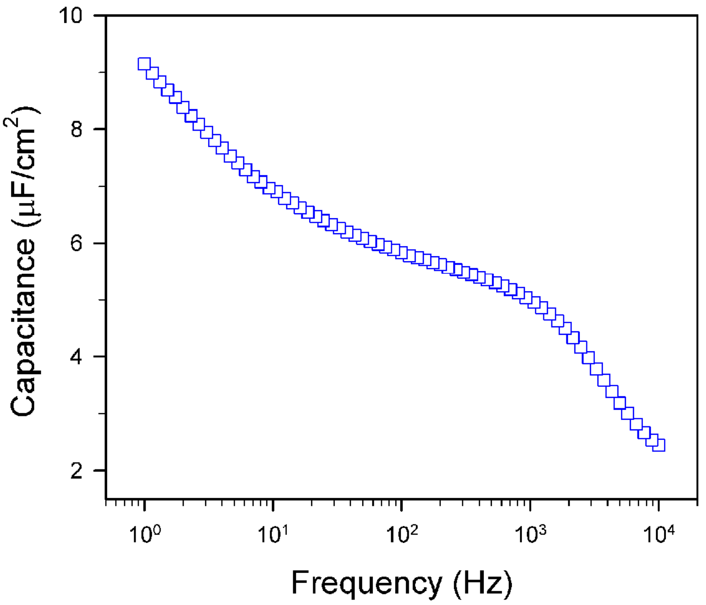

Figure S9 | Frequency dependence of specific capacitance for a 11 μm thick ion gel dielectric layer based on P(VDF-HFP) random copolymer and [EMI][TFSA] ionic liquid.

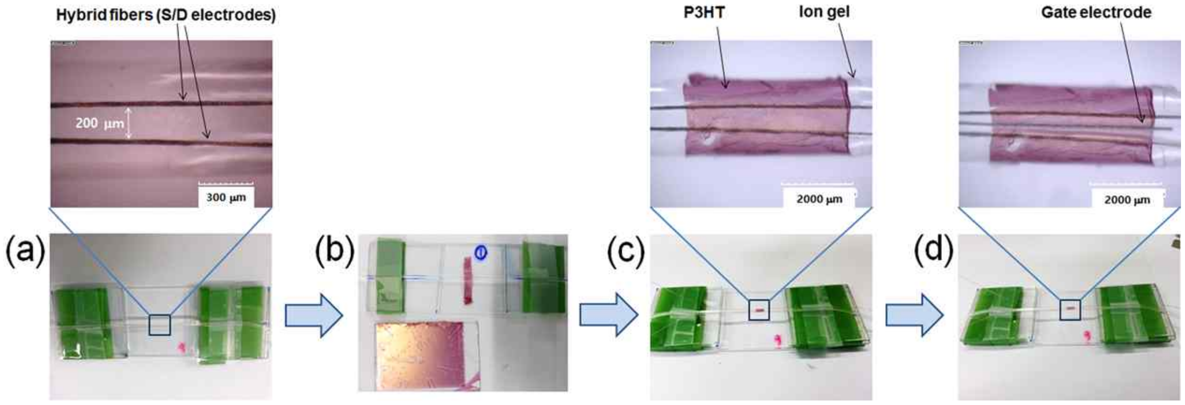

Figure S10 | Digital images of the fiber-type flexible transistor device at different stages during fabrication. (a) Hybrid fiber electrodes (S/D) embedded in a PU monofilament. (b) Ion gel/P3HT double layer transferred onto the S/D electrodes. (c) Cutting the uncovered region of double layer made a complete contact between the S/D electrodes on PU monofilament and P3HT/ion gel double layer. (d) Gate electrode fiber applied onto the ion gel layer and new ion gel layer covered the gate electrode.

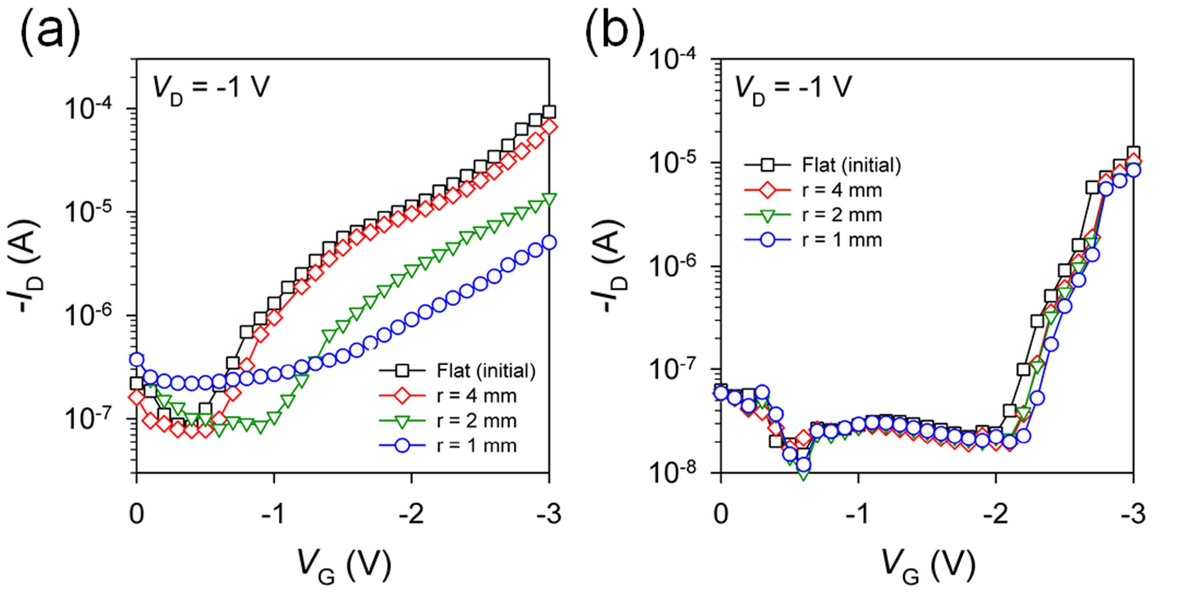

Figure S11 | Changes of transfer curves of the fiber-type transistor devices based on (a) graphene/Ag hybrid fiber (R_{draw} = 1.2) and (b) rGO fiber electrodes obtained at different bending radii.

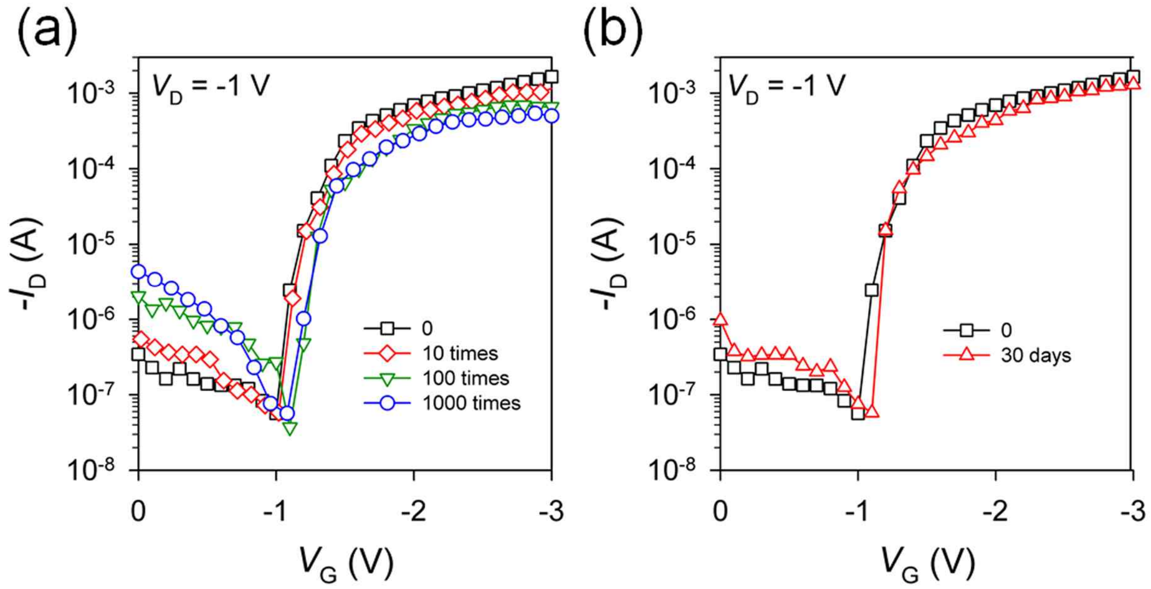

Figure S12 | Changes of transfer curves of the fiber-type transistor devices based on the graphene/Ag hybrid fiber electrodes (R_{draw} = 0.6) (a) during repeated bending cycles at r = 2 mm and (b) being kept in air for 30 days.

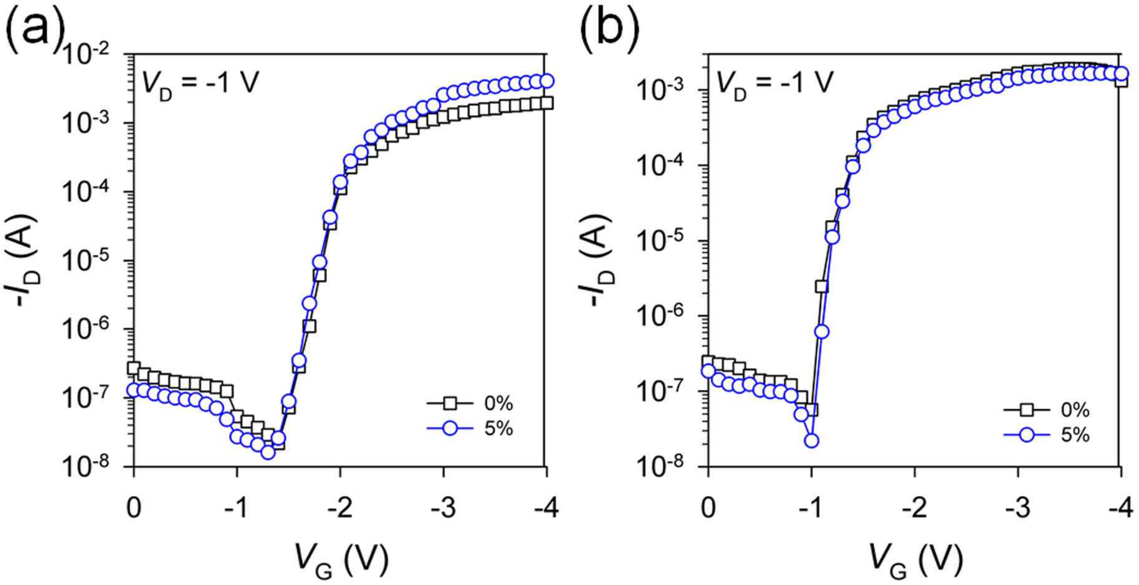

Figure S13 | Changes in the transfer curves of the (a) planar and (b) fiber transistor devices based on the graphene/Ag hybrid fiber electrodes (R_{draw} = 0.6) stretched to 5%.
